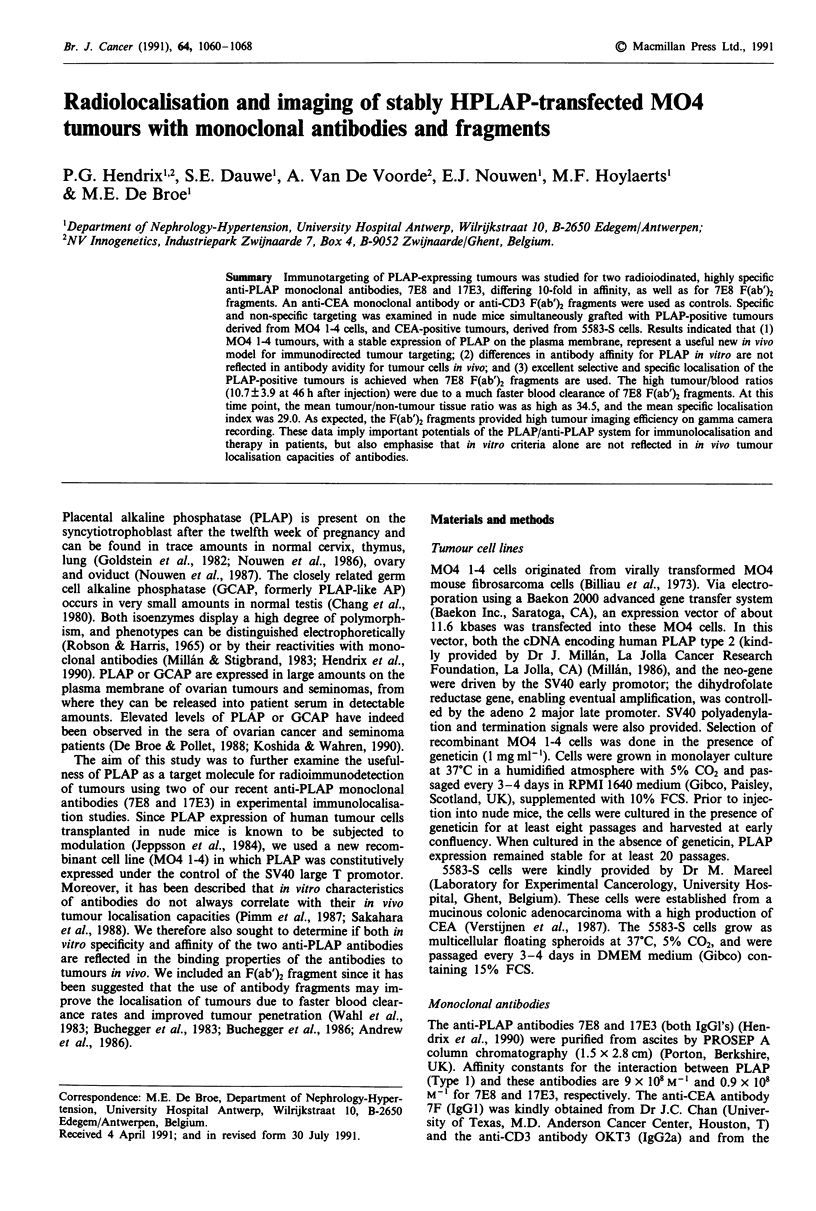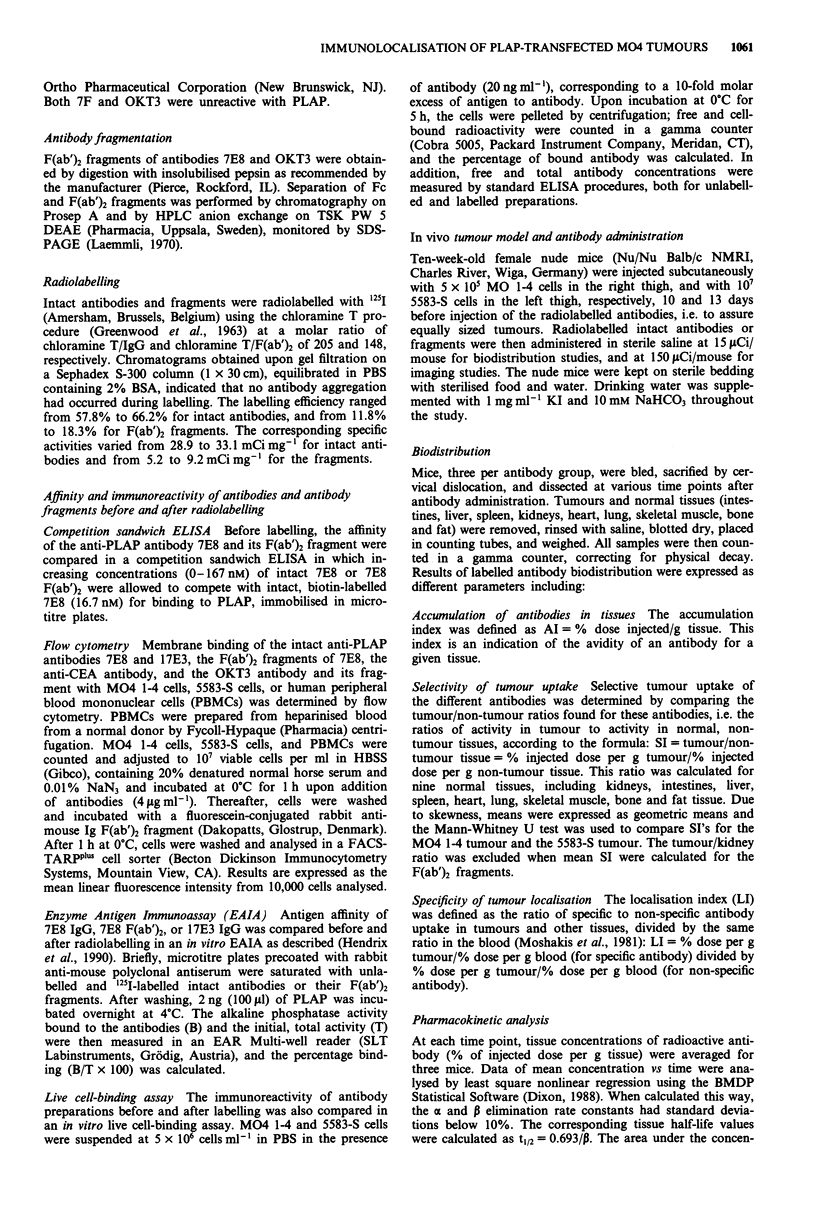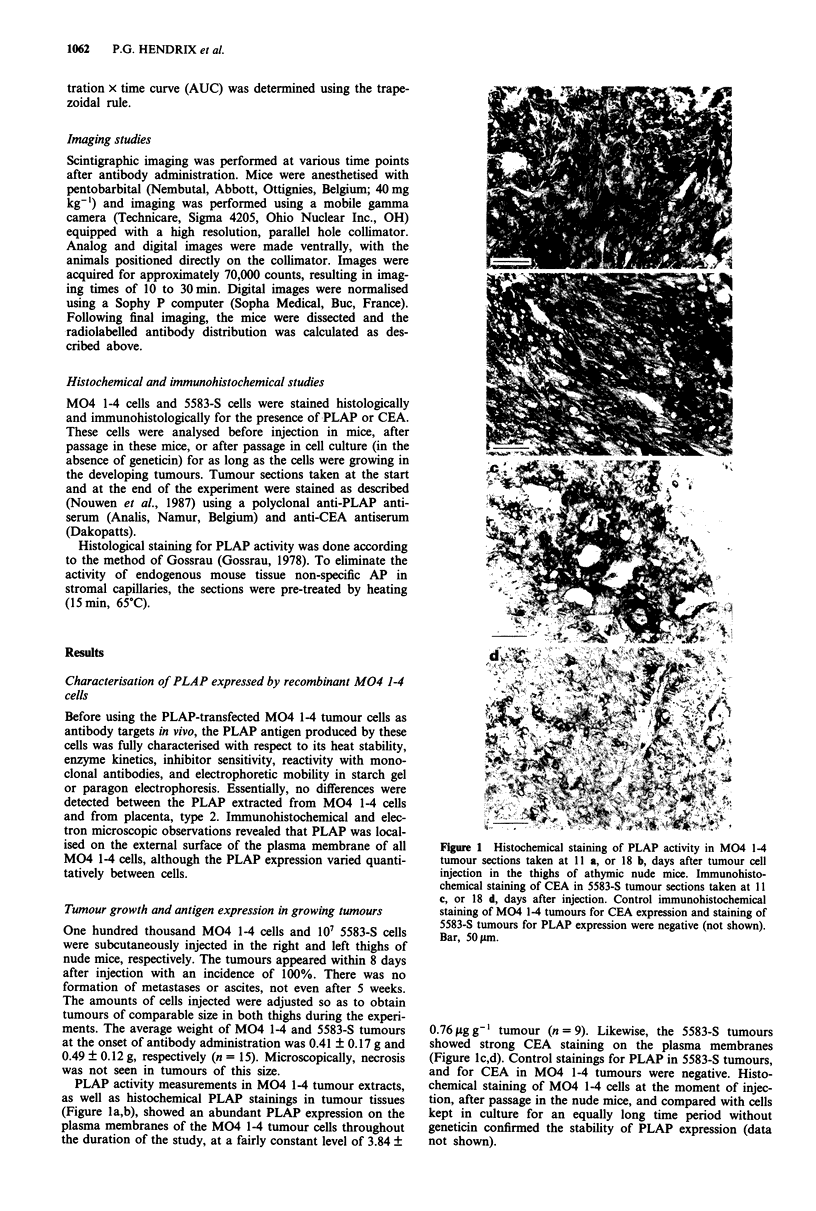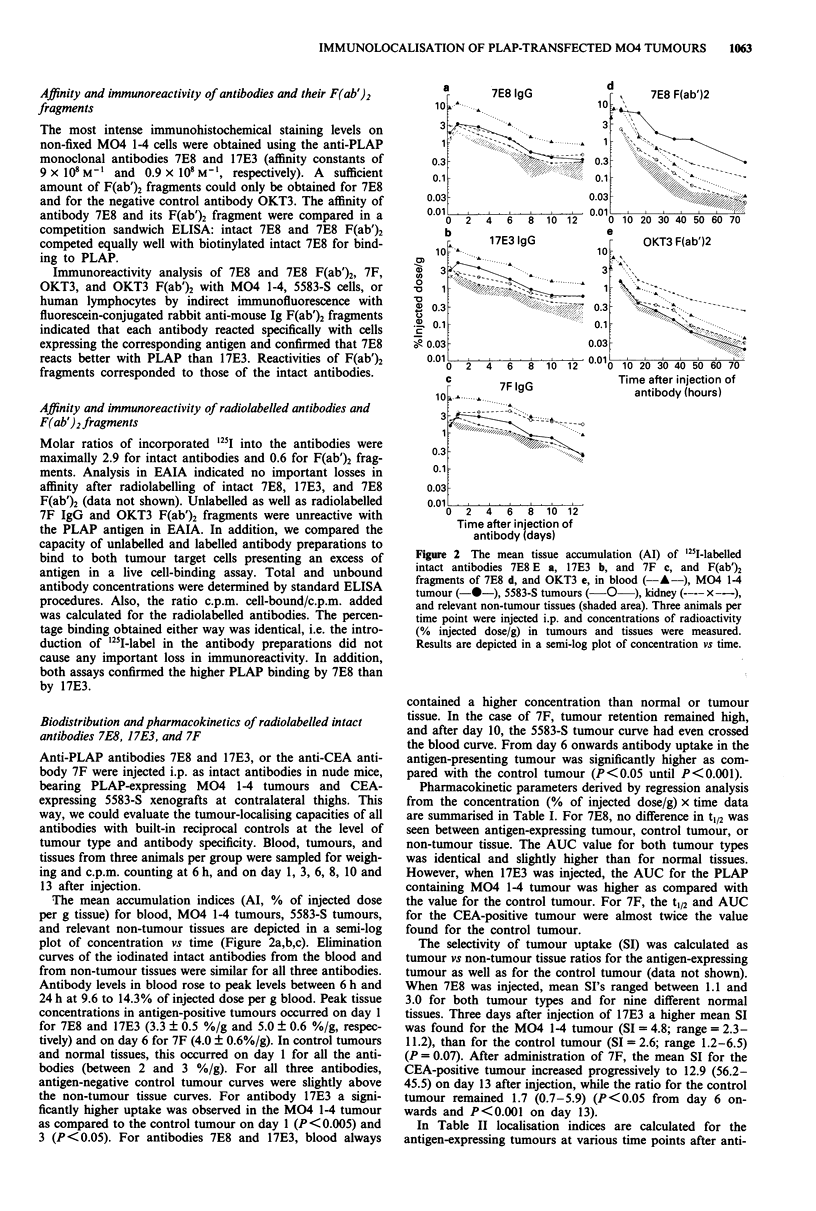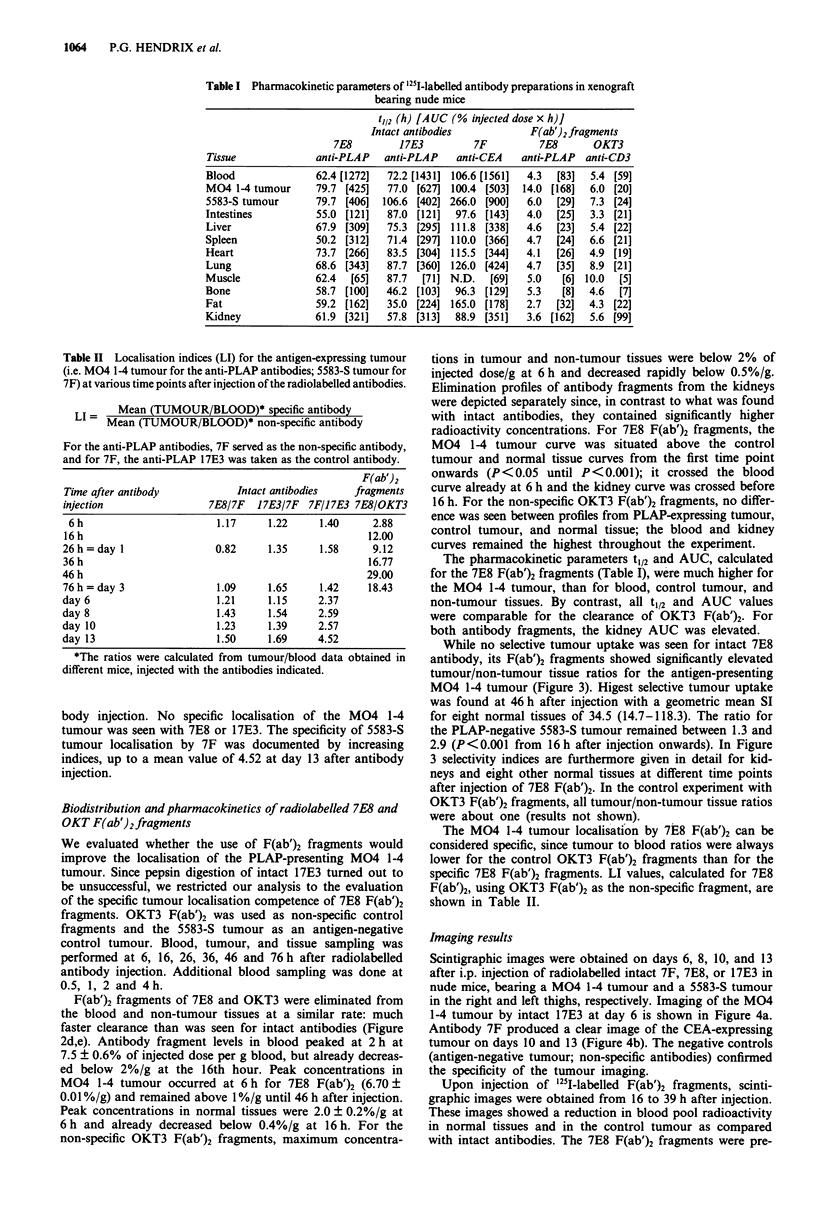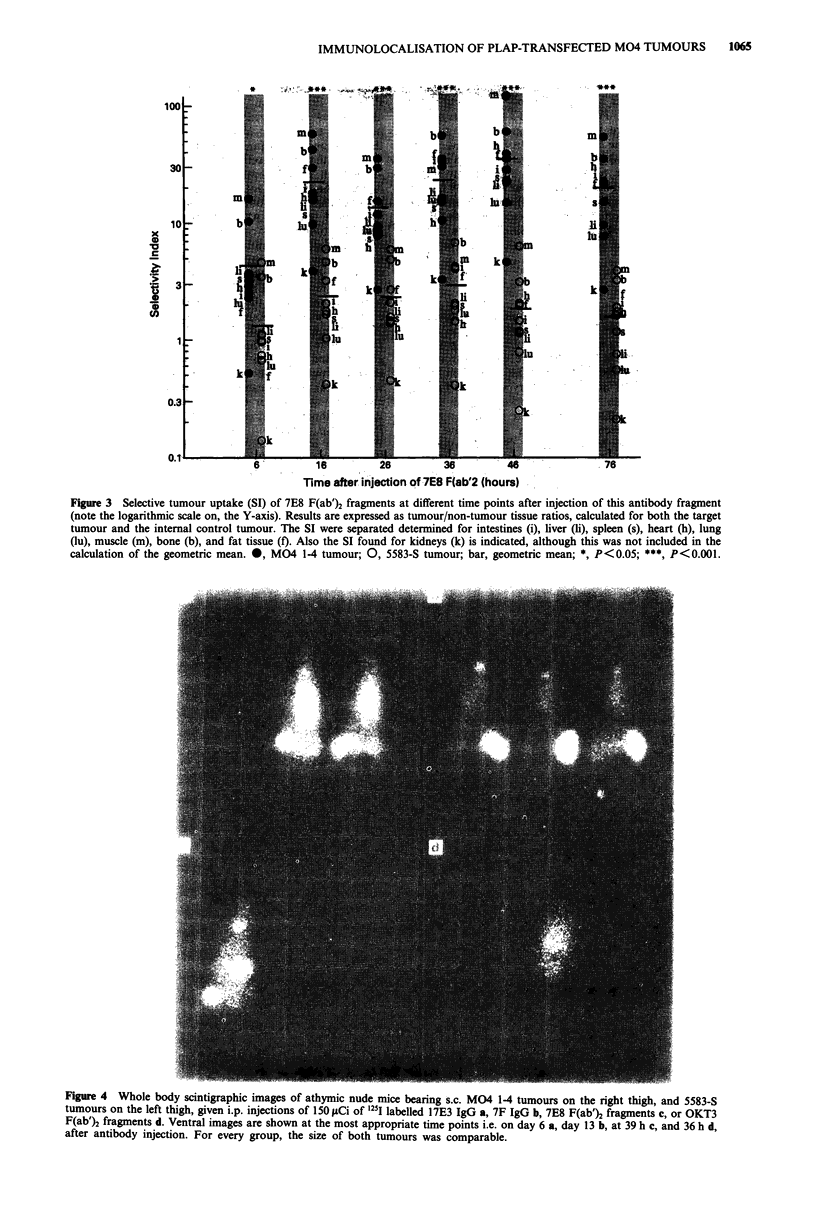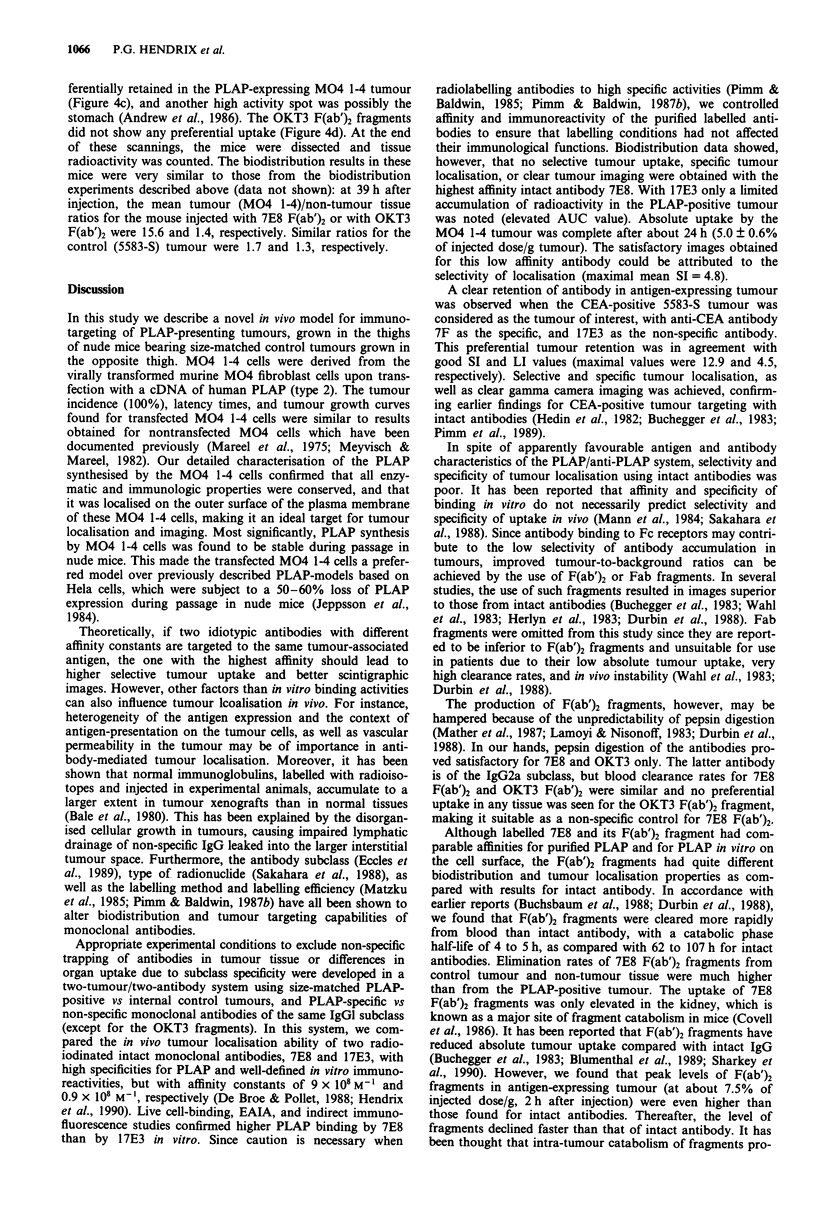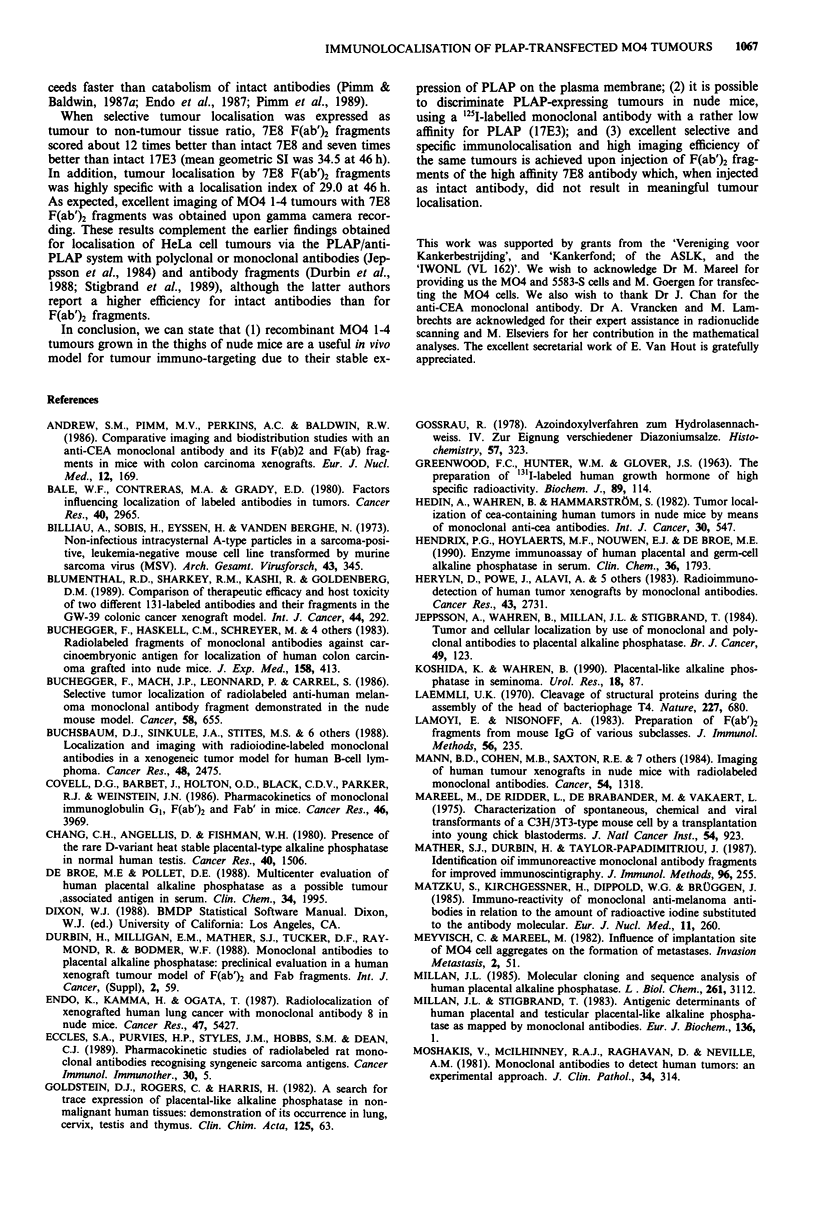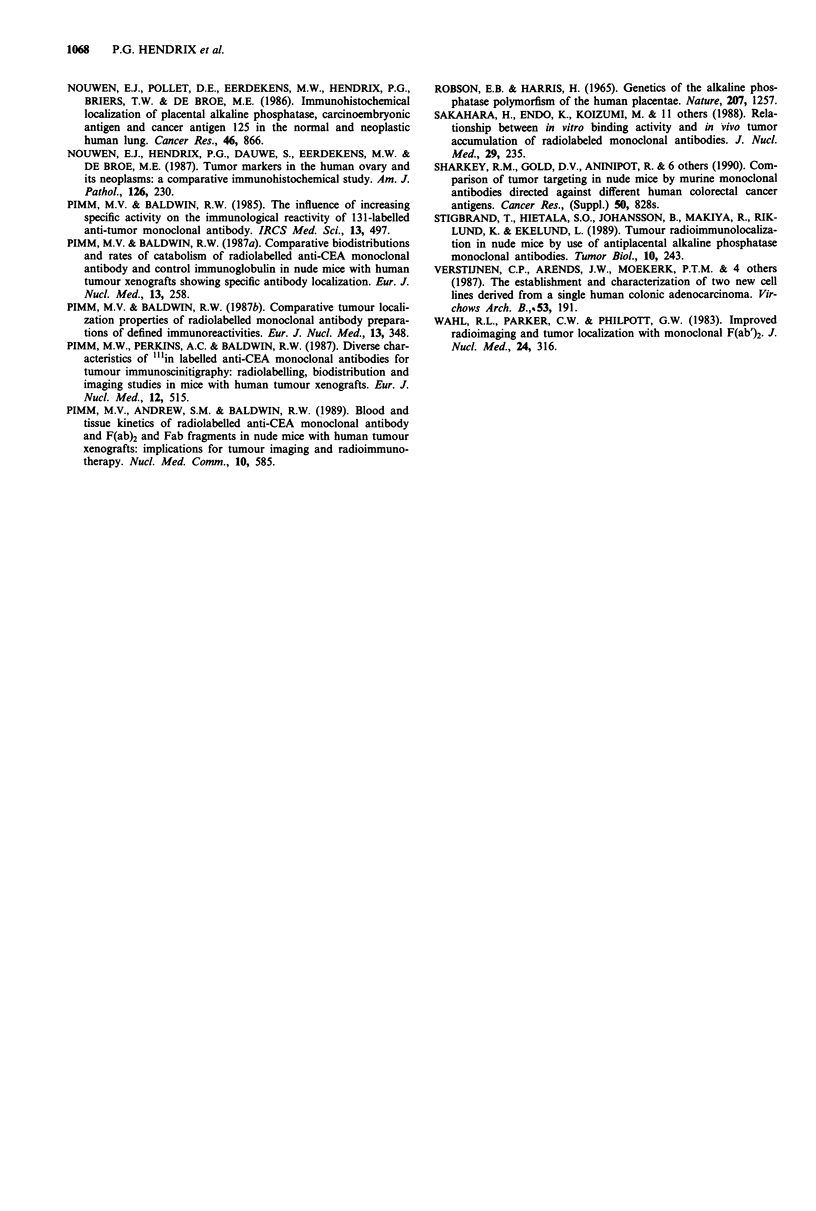# Radiolocalisation and imaging of stably HPLAP-transfected MO4 tumours with monoclonal antibodies and fragments.

**DOI:** 10.1038/bjc.1991.465

**Published:** 1991-12

**Authors:** P. G. Hendrix, S. E. Dauwe, A. Van De Voorde, E. J. Nouwen, M. F. Hoylaerts, M. E. De Broe

**Affiliations:** Department of Nephrology-Hypertension, University Hospital Antwerp, Edegem/Antwerpen, Belgium.

## Abstract

**Images:**


Br. J. Cancer (1991), 64, 1060-1068 © Macmillan Press Ltd., 1991

### Radiolocalisation and imaging of stably HPLAP-transfected MO4 tumours with monoclonal antibodies and fragments

P.G. Hendrix[1,2], S.E. Dauwe[1], A. Van De Voorde[2], E.J. Nouwen[1], M.F. Hoylaerts[1] & M.E. De Broe[1]

[1]*Department of Nephrology-Hypertension, University Hospital Antwerp, Wilrijkstraat 10, B-2650 Edegem/Antwerpen;* [2]*NV Innogenetics, Industriepark Zwijnaarde 7, Box 4, B-9052 Zwijnaarde/Ghent, Belgium.*

**Summary** Immunotargeting of PLAP-expressing tumours was studied for two radioiodinated, highly specific anti-PLAP monoclonal antibodies, 7E8 and 17E3, differing 10-fold in affinity, as well as for 7E8 F(ab')$_2$ fragments. An anti-CEA monoclonal antibody or anti-CD3 F(ab')$_2$ fragments were used as controls. Specific and non-specific targeting was examined in nude mice simultaneously grafted with PLAP-positive tumours derived from MO4 1-4 cells, and CEA-positive tumours, derived from 5583-S cells. Results indicated that (1) MO4 1-4 tumours, with a stable expression of PLAP on the plasma membrane, represent a useful new *in vivo* model for immunodirected tumour targeting; (2) differences in antibody affinity for PLAP *in vitro* are not reflected in antibody avidity for tumour cells *in vivo*; and (3) excellent selective and specific localisation of the PLAP-positive tumours is achieved when 7E8 F(ab')$_2$ fragments are used. The high tumour/blood ratios (10.7 ± 3.9 at 46 h after injection) were due to a much faster blood clearance of 7E8 F(ab')$_2$ fragments. At this time point, the mean tumour/non-tumour tissue ratio was as high as 34.5, and the mean specific localisation index was 29.0. As expected, the F(ab')$_2$ fragments provided high tumour imaging efficiency on gamma camera recording. These data imply important potentials of the PLAP/anti-PLAP system for immunolocalisation and therapy in patients, but also emphasise that *in vitro* criteria alone are not reflected in *in vivo* tumour localisation capacities of antibodies.

Placental alkaline phosphatase (PLAP) is present on the syncytiotrophoblast after the twelfth week of pregnancy and can be found in trace amounts in normal cervix, thymus, lung (Goldstein *et al.*, 1982; Nouwen *et al.*, 1986), ovary and oviduct (Nouwen *et al.*, 1987). The closely related germ cell alkaline phosphatase (GCAP, formerly PLAP-like AP) occurs in very small amounts in normal testis (Chang *et al.*, 1980). Both isoenzymes display a high degree of polymorphism, and phenotypes can be distinguished electrophoretically (Robson & Harris, 1965) or by their reactivities with monoclonal antibodies (Millán & Stigbrand, 1983; Hendrix *et al.*, 1990). PLAP or GCAP are expressed in large amounts on the plasma membrane of ovarian tumours and seminomas, from where they can be released into patient serum in detectable amounts. Elevated levels of PLAP or GCAP have indeed been observed in the sera of ovarian cancer and seminoma patients (De Broe & Pollet, 1988; Koshida & Wahren, 1990).

The aim of this study was to further examine the usefulness of PLAP as a target molecule for radioimmunodetection of tumours using two of our recent anti-PLAP monoclonal antibodies (7E8 and 17E3) in experimental immunolocalisation studies. Since PLAP expression of human tumour cells transplanted in nude mice is known to be subjected to modulation (Jeppsson *et al.*, 1984), we used a new recombinant cell line (MO4 1-4) in which PLAP was constitutively expressed under the control of the SV40 large T promotor. Moreover, it has been described that *in vitro* characteristics of antibodies do not always correlate with their *in vivo* tumour localisation capacities (Pimm *et al.*, 1987; Sakahara *et al.*, 1988). We therefore also sought to determine if both *in vitro* specificity and affinity of the two anti-PLAP antibodies are reflected in the binding properties of the antibodies to tumours *in vivo*. We included an F(ab')$_2$ fragment since it has been suggested that the use of antibody fragments may improve the localisation of tumours due to faster blood clearance rates and improved tumour penetration (Wahl *et al.*, 1983; Buchegger *et al.*, 1983; Buchegger *et al.*, 1986; Andrew *et al.*, 1986).

Correspondence: M.E. De Broe, Department of Nephrology-Hypertension, University Hospital Antwerp, Wilrijkstraat 10, B-2650 Edegem/Antwerpen, Belgium.
Received 4 April 1991; and in revised form 30 July 1991.

### Materials and methods

### *Tumour cell lines*

MO4 1-4 cells originated from virally transformed MO4 mouse fibrosarcoma cells (Billiau *et al.*, 1973). Via electroporation using a Baekon 2000 advanced gene transfer system (Baekon Inc., Saratoga, CA), an expression vector of about 11.6 kbases was transfected into these MO4 cells. In this vector, both the cDNA encoding human PLAP type 2 (kindly provided by Dr J. Millán, La Jolla Cancer Research Foundation, La Jolla, CA) (Millán, 1986), and the neo-gene were driven by the SV40 early promotor; the dihydrofolate reductase gene, enabling eventual amplification, was controlled by the adeno 2 major late promoter. SV40 polyadenylation and termination signals were also provided. Selection of recombinant MO4 1-4 cells was done in the presence of geneticin (1 mg ml$^{-1}$). Cells were grown in monolayer culture at 37°C in a humidified atmosphere with 5% $CO_2$ and passaged every 3-4 days in RPMI 1640 medium (Gibco, Paisley, Scotland, UK), supplemented with 10% FCS. Prior to injection into nude mice, the cells were cultured in the presence of geneticin for at least eight passages and harvested at early confluency. When cultured in the absence of geneticin, PLAP expression remained stable for at least 20 passages.

5583-S cells were kindly provided by Dr M. Mareel (Laboratory for Experimental Cancerology, University Hospital, Ghent, Belgium). These cells were established from a mucinous colonic adenocarcinoma with a high production of CEA (Verstijnen *et al.*, 1987). The 5583-S cells grow as multicellular floating spheroids at 37°C, 5% $CO_2$, and were passaged every 3-4 days in DMEM medium (Gibco) containing 15% FCS.

### *Monoclonal antibodies*

The anti-PLAP antibodies 7E8 and 17E3 (both IgGl's) (Hendrix *et al.*, 1990) were purified from ascites by PROSEP A column chromatography (1.5 × 2.8 cm) (Porton, Berkshire, UK). Affinity constants for the interaction between PLAP (Type 1) and these antibodies are $9 \times 10^8$ M$^{-1}$ and $0.9 \times 10^8$ M$^{-1}$ for 7E8 and 17E3, respectively. The anti-CEA antibody 7F (IgG1) was kindly obtained from Dr J.C. Chan (University of Texas, M.D. Anderson Cancer Center, Houston, T) and the anti-CD3 antibody OKT3 (IgG2a) and from the

Ortho Pharmaceutical Corporation (New Brunswick, NJ). Both 7F and OKT3 were unreactive with PLAP.

*Antibody fragmentation*

$F(ab')_2$ fragments of antibodies 7E8 and OKT3 were obtained by digestion with insolubilised pepsin as recommended by the manufacturer (Pierce, Rockford, IL). Separation of Fc and $F(ab')_2$ fragments was performed by chromatography on Prosep A and by HPLC anion exchange on TSK PW 5 DEAE (Pharmacia, Uppsala, Sweden), monitored by SDS-PAGE (Laemmli, 1970).

*Radiolabelling*

Intact antibodies and fragments were radiolabelled with $^{125}I$ (Amersham, Brussels, Belgium) using the chloramine T procedure (Greenwood *et al.*, 1963) at a molar ratio of chloramine T/IgG and chloramine T/$F(ab')_2$ of 205 and 148, respectively. Chromatograms obtained upon gel filtration on a Sephadex S-300 column (1 × 30 cm), equilibrated in PBS containing 2% BSA, indicated that no antibody aggregation had occurred during labelling. The labelling efficiency ranged from 57.8% to 66.2% for intact antibodies, and from 11.8% to 18.3% for $F(ab')_2$ fragments. The corresponding specific activities varied from 28.9 to 33.1 mCi $mg^{-1}$ for intact antibodies and from 5.2 to 9.2 mCi $mg^{-1}$ for the fragments.

*Affinity and immunoreactivity of antibodies and antibody fragments before and after radiolabelling*

*Competition sandwich ELISA* Before labelling, the affinity of the anti-PLAP antibody 7E8 and its $F(ab')_2$ fragment were compared in a competition sandwich ELISA in which increasing concentrations (0–167 nM) of intact 7E8 or 7E8 $F(ab')_2$ were allowed to compete with intact, biotin-labelled 7E8 (16.7 nM) for binding to PLAP, immobilised in microtitre plates.

*Flow cytometry* Membrane binding of the intact anti-PLAP antibodies 7E8 and 17E3, the $F(ab')_2$ fragments of 7E8, the anti-CEA antibody, and the OKT3 antibody and its fragment with MO4 1-4 cells, 5583-S cells, or human peripheral blood mononuclear cells (PBMCs) was determined by flow cytometry. PBMCs were prepared from heparinised blood from a normal donor by Fycoll-Hypaque (Pharmacia) centrifugation. MO4 1-4 cells, 5583-S cells, and PBMCs were counted and adjusted to $10^7$ viable cells per ml in HBSS (Gibco), containing 20% denatured normal horse serum and 0.01% $NaN_3$ and incubated at 0°C for 1 h upon addition of antibodies (4 μg $ml^{-1}$). Thereafter, cells were washed and incubated with a fluorescein-conjugated rabbit anti-mouse Ig $F(ab')_2$ fragment (Dakopatts, Glostrup, Denmark). After 1 h at 0°C, cells were washed and analysed in a FACS-TARP$^{plus}$ cell sorter (Becton Dickinson Immunocytometry Systems, Mountain View, CA). Results are expressed as the mean linear fluorescence intensity from 10,000 cells analysed.

*Enzyme Antigen Immunoassay (EAIA)* Antigen affinity of 7E8 IgG, 7E8 $F(ab')_2$, or 17E3 IgG was compared before and after radiolabelling in an *in vitro* EAIA as described (Hendrix *et al.*, 1990). Briefly, microtitre plates precoated with rabbit anti-mouse polyclonal antiserum were saturated with unlabelled and $^{125}I$-labelled intact antibodies or their $F(ab')_2$ fragments. After washing, 2 ng (100 μl) of PLAP was incubated overnight at 4°C. The alkaline phosphatase activity bound to the antibodies (B) and the initial, total activity (T) were then measured in an EAR Multi-well reader (SLT Labinstruments, Grödig, Austria), and the percentage binding (B/T × 100) was calculated.

*Live cell-binding assay* The immunoreactivity of antibody preparations before and after labelling was also compared in an *in vitro* live cell-binding assay. MO4 1-4 and 5583-S cells were suspended at 5 × $10^6$ cells $ml^{-1}$ in PBS in the presence of antibody (20 ng $ml^{-1}$), corresponding to a 10-fold molar excess of antigen to antibody. Upon incubation at 0°C for 5 h, the cells were pelleted by centrifugation; free and cell-bound radioactivity were counted in a gamma counter (Cobra 5005, Packard Instrument Company, Meridan, CT), and the percentage of bound antibody was calculated. In addition, free and total antibody concentrations were measured by standard ELISA procedures, both for unlabelled and labelled preparations.

In vivo *tumour model and antibody administration*

Ten-week-old female nude mice (Nu/Nu Balb/c NMRI, Charles River, Wiga, Germany) were injected subcutaneously with 5 × $10^5$ MO 1-4 cells in the right thigh, and with $10^7$ 5583-S cells in the left thigh, respectively, 10 and 13 days before injection of the radiolabelled antibodies, i.e. to assure equally sized tumours. Radiolabelled intact antibodies or fragments were then administered in sterile saline at 15 μCi/mouse for biodistribution studies, and at 150 μCi/mouse for imaging studies. The nude mice were kept on sterile bedding with sterilised food and water. Drinking water was supplemented with 1 mg $ml^{-1}$ KI and 10 mM $NaHCO_3$ throughout the study.

*Biodistribution*

Mice, three per antibody group, were bled, sacrified by cervical dislocation, and dissected at various time points after antibody administration. Tumours and normal tissues (intestines, liver, spleen, kidneys, heart, lung, skeletal muscle, bone and fat) were removed, rinsed with saline, blotted dry, placed in counting tubes, and weighed. All samples were then counted in a gamma counter, correcting for physical decay. Results of labelled antibody biodistribution were expressed as different parameters including:

*Accumulation of antibodies in tissues* The accumulation index was defined as AI = % dose injected/g tissue. This index is an indication of the avidity of an antibody for a given tissue.

*Selectivity of tumour uptake* Selective tumour uptake of the different antibodies was determined by comparing the tumour/non-tumour ratios found for these antibodies, i.e. the ratios of activity in tumour to activity in normal, non-tumour tissues, according to the formula: SI = tumour/non-tumour tissue = % injected dose per g tumour/% injected dose per g non-tumour tissue. This ratio was calculated for nine normal tissues, including kidneys, intestines, liver, spleen, heart, lung, skeletal muscle, bone and fat tissue. Due to skewness, means were expressed as geometric means and the Mann-Whitney U test was used to compare SI's for the MO4 1-4 tumour and the 5583-S tumour. The tumour/kidney ratio was excluded when mean SI were calculated for the $F(ab')_2$ fragments.

*Specificity of tumour localisation* The localisation index (LI) was defined as the ratio of specific to non-specific antibody uptake in tumours and other tissues, divided by the same ratio in the blood (Moshakis *et al.*, 1981): LI = % dose per g tumour/% dose per g blood (for specific antibody) divided by % dose per g tumour/% dose per g blood (for non-specific antibody).

*Pharmacokinetic analysis*

At each time point, tissue concentrations of radioactive antibody (% of injected dose per g tissue) were averaged for three mice. Data of mean concentration *vs* time were analysed by least square nonlinear regression using the BMDP Statistical Software (Dixon, 1988). When calculated this way, the α and β elimination rate constants had standard deviations below 10%. The corresponding tissue half-life values were calculated as $t_{1/2} = 0.693/\beta$. The area under the concentration × time curve (AUC) was determined using the trapezoidal rule.

*Imaging studies*

Scintigraphic imaging was performed at various time points after antibody administration. Mice were anesthetised with pentobarbital (Nembutal, Abbott, Ottignies, Belgium; 40 mg $kg^{-1}$) and imaging was performed using a mobile gamma camera (Technicare, Sigma 4205, Ohio Nuclear Inc., OH) equipped with a high resolution, parallel hole collimator. Analog and digital images were made ventrally, with the animals positioned directly on the collimator. Images were acquired for approximately 70,000 counts, resulting in imaging times of 10 to 30 min. Digital images were normalised using a Sophy P computer (Sopha Medical, Buc, France). Following final imaging, the mice were dissected and the radiolabelled antibody distribution was calculated as described above.

*Histochemical and immunohistochemical studies*

MO4 1-4 cells and 5583-S cells were stained histologically and immunohistologically for the presence of PLAP or CEA. These cells were analysed before injection in mice, after passage in these mice, or after passage in cell culture (in the absence of geneticin) for as long as the cells were growing in the developing tumours. Tumour sections taken at the start and at the end of the experiment were stained as described (Nouwen *et al.*, 1987) using a polyclonal anti-PLAP antiserum (Analis, Namur, Belgium) and anti-CEA antiserum (Dakopatts).

Histological staining for PLAP activity was done according to the method of Gossrau (Gossrau, 1978). To eliminate the activity of endogenous mouse tissue non-specific AP in stromal capillaries, the sections were pre-treated by heating (15 min, 65°C).

### Results

*Characterisation of PLAP expressed by recombinant MO4 1-4 cells*

Before using the PLAP-transfected MO4 1-4 tumour cells as antibody targets *in vivo*, the PLAP antigen produced by these cells was fully characterised with respect to its heat stability, enzyme kinetics, inhibitor sensitivity, reactivity with monoclonal antibodies, and electrophoretic mobility in starch gel or paragon electrophoresis. Essentially, no differences were detected between the PLAP extracted from MO4 1-4 cells and from placenta, type 2. Immunohistochemical and electron microscopic observations revealed that PLAP was localised on the external surface of the plasma membrane of all MO4 1-4 cells, although the PLAP expression varied quantitatively between cells.

*Tumour growth and antigen expression in growing tumours*

One hundred thousand MO4 1-4 cells and $10^7$ 5583-S cells were subcutaneously injected in the right and left thighs of nude mice, respectively. The tumours appeared within 8 days after injection with an incidence of 100%. There was no formation of metastases or ascites, not even after 5 weeks. The amounts of cells injected were adjusted so as to obtain tumours of comparable size in both thighs during the experiments. The average weight of MO4 1-4 and 5583-S tumours at the onset of antibody administration was 0.41 ± 0.17 g and 0.49 ± 0.12 g, respectively ($n = 15$). Microscopically, necrosis was not seen in tumours of this size.

PLAP activity measurements in MO4 1-4 tumour extracts, as well as histochemical PLAP stainings in tumour tissues (Figure 1a,b), showed an abundant PLAP expression on the plasma membranes of the MO4 1-4 tumour cells throughout the duration of the study, at a fairly constant level of 3.84 ± 0.76 $\mu g\ g^{-1}$ tumour ($n = 9$). Likewise, the 5583-S tumours showed strong CEA staining on the plasma membranes (Figure 1c,d). Control stainings for PLAP in 5583-S tumours, and for CEA in MO4 1-4 tumours were negative. Histochemical staining of MO4 1-4 cells at the moment of injection, after passage in the nude mice, and compared with cells kept in culture for an equally long time period without geneticin confirmed the stability of PLAP expression (data not shown).

**Figure 1** Histochemical staining of PLAP activity in MO4 1-4 tumour sections taken at 11 **a**, or 18 **b**, days after tumour cell injection in the thighs of athymic nude mice. Immunohistochemical staining of CEA in 5583-S tumour sections taken at 11 **c**, or 18 **d**, days after injection. Control immunohistochemical staining of MO4 1-4 tumours for CEA expression and staining of 5583-S tumours for PLAP expression were negative (not shown). Bar, 50 µm.

### *Affinity and immunoreactivity of antibodies and their $F(ab')_2$ fragments*

The most intense immunohistochemical staining levels on non-fixed MO4 1-4 cells were obtained using the anti-PLAP monoclonal antibodies 7E8 and 17E3 (affinity constants of $9 \times 10^8\,M^{-1}$ and $0.9 \times 10^8\,M^{-1}$, respectively). A sufficient amount of $F(ab')_2$ fragments could only be obtained for 7E8 and for the negative control antibody OKT3. The affinity of antibody 7E8 and its $F(ab')_2$ fragment were compared in a competition sandwich ELISA: intact 7E8 and 7E8 $F(ab')_2$ competed equally well with biotinylated intact 7E8 for binding to PLAP.

Immunoreactivity analysis of 7E8 and 7E8 $F(ab')_2$, 7F, OKT3, and OKT3 $F(ab')_2$ with MO4 1-4, 5583-S cells, or human lymphocytes by indirect immunofluorescence with fluorescein-conjugated rabbit anti-mouse Ig $F(ab')_2$ fragments indicated that each antibody reacted specifically with cells expressing the corresponding antigen and confirmed that 7E8 reacts better with PLAP than 17E3. Reactivities of $F(ab')_2$ fragments corresponded to those of the intact antibodies.

### *Affinity and immunoreactivity of radiolabelled antibodies and $F(ab')_2$ fragments*

Molar ratios of incorporated $^{125}I$ into the antibodies were maximally 2.9 for intact antibodies and 0.6 for $F(ab')_2$ fragments. Analysis in EAIA indicated no important losses in affinity after radiolabelling of intact 7E8, 17E3, and 7E8 $F(ab')_2$ (data not shown). Unlabelled as well as radiolabelled 7F IgG and OKT3 $F(ab')_2$ fragments were unreactive with the PLAP antigen in EAIA. In addition, we compared the capacity of unlabelled and labelled antibody preparations to bind to both tumour target cells presenting an excess of antigen in a live cell-binding assay. Total and unbound antibody concentrations were determined by standard ELISA procedures. Also, the ratio c.p.m. cell-bound/c.p.m. added was calculated for the radiolabelled antibodies. The percentage binding obtained either way was identical, i.e. the introduction of $^{125}I$-label in the antibody preparations did not cause any important loss in immunoreactivity. In addition, both assays confirmed the higher PLAP binding by 7E8 than by 17E3.

### *Biodistribution and pharmacokinetics of radiolabelled intact antibodies 7E8, 17E3, and 7F*

Anti-PLAP antibodies 7E8 and 17E3, or the anti-CEA antibody 7F were injected i.p. as intact antibodies in nude mice, bearing PLAP-expressing MO4 1-4 tumours and CEA-expressing 5583-S xenografts at contralateral thighs. This way, we could evaluate the tumour-localising capacities of all antibodies with built-in reciprocal controls at the level of tumour type and antibody specificity. Blood, tumours, and tissues from three animals per group were sampled for weighing and c.p.m. counting at 6 h, and on day 1, 3, 6, 8, 10 and 13 after injection.

The mean accumulation indices (AI, % of injected dose per g tissue) for blood, MO4 1-4 tumours, 5583-S tumours, and relevant non-tumour tissues are depicted in a semi-log plot of concentration *vs* time (Figure 2a,b,c). Elimination curves of the iodinated intact antibodies from the blood and from non-tumour tissues were similar for all three antibodies. Antibody levels in blood rose to peak levels between 6 h and 24 h at 9.6 to 14.3% of injected dose per g blood. Peak tissue concentrations in antigen-positive tumours occurred on day 1 for 7E8 and 17E3 ($3.3 \pm 0.5$ %/g and $5.0 \pm 0.6$ %/g, respectively) and on day 6 for 7F ($4.0 \pm 0.6$%/g). In control tumours and normal tissues, this occurred on day 1 for all the antibodies (between 2 and 3 %/g). For all three antibodies, antigen-negative control tumour curves were slightly above the non-tumour tissue curves. For antibody 17E3 a significantly higher uptake was observed in the MO4 1-4 tumour as compared to the control tumour on day 1 ($P < 0.005$) and 3 ($P < 0.05$). For antibodies 7E8 and 17E3, blood always

**Figure 2** The mean tissue accumulation (AI) of $^{125}I$-labelled intact antibodies 7E8 E **a**, 17E3 **b**, and 7F **c**, and $F(ab')_2$ fragments of 7E8 **d**, and OKT3 **e**, in blood (—▲—), MO4 1-4 tumour (—●—), 5583-S tumours (—O—), kidney (----×----), and relevant non-tumour tissues (shaded area). Three animals per time point were injected i.p. and concentrations of radioactivity (% injected dose/g) in tumours and tissues were measured. Results are depicted in a semi-log plot of concentration *vs* time.

contained a higher concentration than normal or tumour tissue. In the case of 7F, tumour retention remained high, and after day 10, the 5583-S tumour curve had even crossed the blood curve. From day 6 onwards antibody uptake in the antigen-presenting tumour was significantly higher as compared with the control tumour ($P < 0.05$ until $P < 0.001$).

Pharmacokinetic parameters derived by regression analysis from the concentration (% of injected dose/g) × time data are summarised in Table I. For 7E8, no difference in $t_{1/2}$ was seen between antigen-expressing tumour, control tumour, or non-tumour tissue. The AUC value for both tumour types was identical and slightly higher than for normal tissues. However, when 17E3 was injected, the AUC for the PLAP containing MO4 1-4 tumour was higher as compared with the value for the control tumour. For 7F, the $t_{1/2}$ and AUC for the CEA-positive tumour were almost twice the value found for the control tumour.

The selectivity of tumour uptake (SI) was calculated as tumour *vs* non-tumour tissue ratios for the antigen-expressing tumour as well as for the control tumour (data not shown). When 7E8 was injected, mean SI's ranged between 1.1 and 3.0 for both tumour types and for nine different normal tissues. Three days after injection of 17E3 a higher mean SI was found for the MO4 1-4 tumour (SI = 4.8; range = 2.3–11.2), than for the control tumour (SI = 2.6; range 1.2–6.5) ($P = 0.07$). After administration of 7F, the mean SI for the CEA-positive tumour increased progressively to 12.9 (56.2–45.5) on day 13 after injection, while the ratio for the control tumour remained 1.7 (0.7–5.9) ($P < 0.05$ from day 6 onwards and $P < 0.001$ on day 13).

In Table II localisation indices are calculated for the antigen-expressing tumours at various time points after anti-

**Table I** Pharmacokinetic parameters of $^{125}$I-labelled antibody preparations in xenograft bearing nude mice

| | $t_{1/2}$ (h) [AUC (% injected dose × h)] | | | | |
|---|---|---|---|---|---|
| | Intact antibodies | | | F(ab')$_2$ fragments | |
| Tissue | 7E8 anti-PLAP | 17E3 anti-PLAP | 7F anti-CEA | 7E8 anti-PLAP | OKT3 anti-CD3 |
| Blood | 62.4 [1272] | 72.2 [1431] | 106.6 [1561] | 4.3 [83] | 5.4 [59] |
| MO4 1-4 tumour | 79.7 [425] | 77.0 [627] | 100.4 [503] | 14.0 [168] | 6.0 [20] |
| 5583-S tumour | 79.7 [406] | 106.6 [402] | 266.0 [900] | 6.0 [29] | 7.3 [24] |
| Intestines | 55.0 [121] | 87.0 [121] | 97.6 [143] | 4.0 [25] | 3.3 [21] |
| Liver | 67.9 [309] | 75.3 [295] | 111.8 [338] | 4.6 [23] | 5.4 [22] |
| Spleen | 50.2 [312] | 71.4 [297] | 110.0 [366] | 4.7 [24] | 6.6 [21] |
| Heart | 73.7 [266] | 83.5 [304] | 115.5 [344] | 4.1 [26] | 4.9 [19] |
| Lung | 68.6 [343] | 87.7 [360] | 126.0 [424] | 4.7 [35] | 8.9 [21] |
| Muscle | 62.4 [65] | 87.7 [71] | N.D. [69] | 5.0 [6] | 10.0 [5] |
| Bone | 58.7 [100] | 46.2 [103] | 96.3 [129] | 5.3 [8] | 4.6 [7] |
| Fat | 59.2 [162] | 35.0 [224] | 165.0 [178] | 2.7 [32] | 4.3 [22] |
| Kidney | 61.9 [321] | 57.8 [313] | 88.9 [351] | 3.6 [162] | 5.6 [99] |

**Table II** Localisation indices (LI) for the antigen-expressing tumour (i.e. MO4 1-4 tumour for the anti-PLAP antibodies; 5583-S tumour for 7F) at various time points after injection of the radiolabelled antibodies.

$$LI = \frac{\text{Mean (TUMOUR/BLOOD)* specific antibody}}{\text{Mean (TUMOUR/BLOOD)* non-specific antibody}}$$

For the anti-PLAP antibodies, 7F served as the non-specific antibody, and for 7F, the anti-PLAP 17E3 was taken as the control antibody.

| Time after antibody injection | Intact antibodies 7E8/7F | 17E3/7F | 7F/17E3 | F(ab')$_2$ fragments 7E8/OKT3 |
|---|---|---|---|---|
| 6 h | 1.17 | 1.22 | 1.40 | 2.88 |
| 16 h | | | | 12.00 |
| 26 h = day 1 | 0.82 | 1.35 | 1.58 | 9.12 |
| 36 h | | | | 16.77 |
| 46 h | | | | 29.00 |
| 76 h = day 3 | 1.09 | 1.65 | 1.42 | 18.43 |
| day 6 | 1.21 | 1.15 | 2.37 | |
| day 8 | 1.43 | 1.54 | 2.59 | |
| day 10 | 1.23 | 1.39 | 2.57 | |
| day 13 | 1.50 | 1.69 | 4.52 | |

*The ratios were calculated from tumour/blood data obtained in different mice, injected with the antibodies indicated.

body injection. No specific localisation of the MO4 1-4 tumour was seen with 7E8 or 17E3. The specificity of 5583-S tumour localisation by 7F was documented by increasing indices, up to a mean value of 4.52 at day 13 after antibody injection.

### *Biodistribution and pharmacokinetics of radiolabelled 7E8 and OKT F(ab')$_2$ fragments*

We evaluated whether the use of F(ab')$_2$ fragments would improve the localisation of the PLAP-presenting MO4 1-4 tumour. Since pepsin digestion of intact 17E3 turned out to be unsuccessful, we restricted our analysis to the evaluation of the specific tumour localisation competence of 7E8 F(ab')$_2$ fragments. OKT3 F(ab')$_2$ was used as non-specific control fragments and the 5583-S tumour as an antigen-negative control tumour. Blood, tumour, and tissue sampling was performed at 6, 16, 26, 36, 46 and 76 h after radiolabelled antibody injection. Additional blood sampling was done at 0.5, 1, 2 and 4 h.

F(ab')$_2$ fragments of 7E8 and OKT3 were eliminated from the blood and non-tumour tissues at a similar rate: much faster clearance than was seen for intact antibodies (Figure 2d,e). Antibody fragment levels in blood peaked at 2 h at 7.5 ± 0.6% of injected dose per g blood, but already decreased below 2%/g at the 16th hour. Peak concentrations in MO4 1-4 tumour occurred at 6 h for 7E8 F(ab')$_2$ (6.70 ± 0.01%/g) and remained above 1%/g until 46 h after injection. Peak concentrations in normal tissues were 2.0 ± 0.2%/g at 6 h and already decreased below 0.4%/g at 16 h. For the non-specific OKT3 F(ab')$_2$ fragments, maximum concentrations in tumour and non-tumour tissues were below 2% of injected dose/g at 6 h and decreased rapidly below 0.5%/g. Elimination profiles of antibody fragments from the kidneys were depicted separately since, in contrast to what was found with intact antibodies, they contained significantly higher radioactivity concentrations. For 7E8 F(ab')$_2$ fragments, the MO4 1-4 tumour curve was situated above the control tumour and normal tissue curves from the first time point onwards ($P<0.05$ until $P<0.001$); it crossed the blood curve already at 6 h and the kidney curve was crossed before 16 h. For the non-specific OKT3 F(ab')$_2$ fragments, no difference was seen between profiles from PLAP-expressing tumour, control tumour, and normal tissue; the blood and kidney curves remained the highest throughout the experiment.

The pharmacokinetic parameters $t_{1/2}$ and AUC, calculated for the 7E8 F(ab')$_2$ fragments (Table I), were much higher for the MO4 1-4 tumour, than for blood, control tumour, and non-tumour tissues. By contrast, all $t_{1/2}$ and AUC values were comparable for the clearance of OKT3 F(ab')$_2$. For both antibody fragments, the kidney AUC was elevated.

While no selective tumour uptake was seen for intact 7E8 antibody, its F(ab')$_2$ fragments showed significantly elevated tumour/non-tumour tissue ratios for the antigen-presenting MO4 1-4 tumour (Figure 3). Higest selective tumour uptake was found at 46 h after injection with a geometric mean SI for eight normal tissues of 34.5 (14.7–118.3). The ratio for the PLAP-negative 5583-S tumour remained between 1.3 and 2.9 ($P<0.001$ from 16 h after injection onwards). In Figure 3 selectivity indices are furthermore given in detail for kidneys and eight other normal tissues at different time points after injection of 7E8 F(ab')$_2$. In the control experiment with OKT3 F(ab')$_2$ fragments, all tumour/non-tumour tissue ratios were about one (results not shown).

The MO4 1-4 tumour localisation by 7E8 F(ab')$_2$ can be considered specific, since tumour to blood ratios were always lower for the control OKT3 F(ab')$_2$ fragments than for the specific 7E8 F(ab')$_2$ fragments. LI values, calculated for 7E8 F(ab')$_2$, using OKT3 F(ab')$_2$ as the non-specific fragment, are shown in Table II.

### *Imaging results*

Scintigraphic images were obtained on days 6, 8, 10, and 13 after i.p. injection of radiolabelled intact 7F, 7E8, or 17E3 in nude mice, bearing a MO4 1-4 tumour and a 5583-S tumour in the right and left thighs, respectively. Imaging of the MO4 1-4 tumour by intact 17E3 at day 6 is shown in Figure 4a. Antibody 7F produced a clear image of the CEA-expressing tumour on days 10 and 13 (Figure 4b). The negative controls (antigen-negative tumour; non-specific antibodies) confirmed the specificity of the tumour imaging.

Upon injection of $^{125}$I-labelled F(ab')$_2$ fragments, scintigraphic images were obtained from 16 to 39 h after injection. These images showed a reduction in blood pool radioactivity in normal tissues and in the control tumour as compared with intact antibodies. The 7E8 F(ab')$_2$ fragments were pre-

**Figure 3** Selective tumour uptake (SI) of 7E8 F(ab')$_2$ fragments at different time points after injection of this antibody fragment (note the logarithmic scale on, the Y-axis). Results are expressed as tumour/non-tumour tissue ratios, calculated for both the target tumour and the internal control tumour. The SI were separated determined for intestines (i), liver (li), spleen (s), heart (h), lung (lu), muscle (m), bone (b), and fat tissue (f). Also the SI found for kidneys (k) is indicated, although this was not included in the calculation of the geometric mean. ●, MO4 1-4 tumour; O, 5583-S tumour; bar, geometric mean; *, $P<0.05$; ***, $P<0.001$.

**Figure 4** Whole body scintigraphic images of athymic nude mice bearing s.c. MO4 1-4 tumours on the right thigh, and 5583-S tumours on the left thigh, given i.p. injections of 150 µCi of $^{125}I$ labelled 17E3 IgG **a**, 7F IgG **b**, 7E8 F(ab')$_2$ fragments **c**, or OKT3 F(ab')$_2$ fragments **d**. Ventral images are shown at the most appropriate time points i.e. on day 6 **a**, day 13 **b**, at 39 h **c**, and 36 h **d**, after antibody injection. For every group, the size of both tumours was comparable.

ferentially retained in the PLAP-expressing MO4 1-4 tumour (Figure 4c), and another high activity spot was possibly the stomach (Andrew *et al.*, 1986). The OKT3 F(ab')$_2$ fragments did not show any preferential uptake (Figure 4d). At the end of these scannings, the mice were dissected and tissue radioactivity was counted. The biodistribution results in these mice were very similar to those from the biodistribution experiments described above (data not shown): at 39 h after injection, the mean tumour (MO4 1-4)/non-tumour tissue ratios for the mouse injected with 7E8 F(ab')$_2$ or with OKT3 F(ab')$_2$ were 15.6 and 1.4, respectively. Similar ratios for the control (5583-S) tumour were 1.7 and 1.3, respectively.

### Discussion

In this study we describe a novel *in vivo* model for immunotargeting of PLAP-presenting tumours, grown in the thighs of nude mice bearing size-matched control tumours grown in the opposite thigh. MO4 1-4 cells were derived from the virally transformed murine MO4 fibroblast cells upon transfection with a cDNA of human PLAP (type 2). The tumour incidence (100%), latency times, and tumour growth curves found for transfected MO4 1-4 cells were similar to results obtained for nontransfected MO4 cells which have been documented previously (Mareel *et al.*, 1975; Meyvisch & Mareel, 1982). Our detailed characterisation of the PLAP synthesised by the MO4 1-4 cells confirmed that all enzymatic and immunologic properties were conserved, and that it was localised on the outer surface of the plasma membrane of these MO4 1-4 cells, making it an ideal target for tumour localisation and imaging. Most significantly, PLAP synthesis by MO4 1-4 cells was found to be stable during passage in nude mice. This made the transfected MO4 1-4 cells a preferred model over previously described PLAP-models based on Hela cells, which were subject to a 50–60% loss of PLAP expression during passage in nude mice (Jeppsson *et al.*, 1984).

Theoretically, if two idiotypic antibodies with different affinity constants are targeted to the same tumour-associated antigen, the one with the highest affinity should lead to higher selective tumour uptake and better scintigraphic images. However, other factors than *in vitro* binding activities can also influence tumour lcoalisation *in vivo*. For instance, heterogeneity of the antigen expression and the context of antigen-presentation on the tumour cells, as well as vascular permeability in the tumour may be of importance in antibody-mediated tumour localisation. Moreover, it has been shown that normal immunoglobulins, labelled with radioisotopes and injected in experimental animals, accumulate to a larger extent in tumour xenografts than in normal tissues (Bale *et al.*, 1980). This has been explained by the disorganised cellular growth in tumours, causing impaired lymphatic drainage of non-specific IgG leaked into the larger interstitial tumour space. Furthermore, the antibody subclass (Eccles *et al.*, 1989), type of radionuclide (Sakahara *et al.*, 1988), as well as the labelling method and labelling efficiency (Matzku *et al.*, 1985; Pimm & Baldwin, 1987*b*) have all been shown to alter biodistribution and tumour targeting capabilities of monoclonal antibodies.

Appropriate experimental conditions to exclude non-specific trapping of antibodies in tumour tissue or differences in organ uptake due to subclass specificity were developed in a two-tumour/two-antibody system using size-matched PLAP-positive *vs* internal control tumours, and PLAP-specific *vs* non-specific monoclonal antibodies of the same IgGl subclass (except for the OKT3 fragments). In this system, we compared the *in vivo* tumour localisation ability of two radioiodinated intact monoclonal antibodies, 7E8 and 17E3, with high specificities for PLAP and well-defined *in vitro* immunoreactivities, but with affinity constants of $9 \times 10^8$ M$^{-1}$ and $0.9 \times 10^8$ M$^{-1}$, respectively (De Broe & Pollet, 1988; Hendrix *et al.*, 1990). Live cell-binding, EAIA, and indirect immunofluorescence studies confirmed higher PLAP binding by 7E8 than by 17E3 *in vitro*. Since caution is necessary when radiolabelling antibodies to high specific activities (Pimm & Baldwin, 1985; Pimm & Baldwin, 1987*b*), we controlled affinity and immunoreactivity of the purified labelled antibodies to ensure that labelling conditions had not affected their immunological functions. Biodistribution data showed, however, that no selective tumour uptake, specific tumour localisation, or clear tumour imaging were obtained with the highest affinity intact antibody 7E8. With 17E3 only a limited accumulation of radioactivity in the PLAP-positive tumour was noted (elevated AUC value). Absolute uptake by the MO4 1-4 tumour was complete after about 24 h (5.0 ± 0.6% of injected dose/g tumour). The satisfactory images obtained for this low affinity antibody could be attributed to the selectivity of localisation (maximal mean SI = 4.8).

A clear retention of antibody in antigen-expressing tumour was observed when the CEA-positive 5583-S tumour was considered as the tumour of interest, with anti-CEA antibody 7F as the specific, and 17E3 as the non-specific antibody. This preferential tumour retention was in agreement with good SI and LI values (maximal values were 12.9 and 4.5, respectively). Selective and specific tumour localisation, as well as clear gamma camera imaging was achieved, confirming earlier findings for CEA-positive tumour targeting with intact antibodies (Hedin *et al.*, 1982; Buchegger *et al.*, 1983; Pimm *et al.*, 1989).

In spite of apparently favourable antigen and antibody characteristics of the PLAP/anti-PLAP system, selectivity and specificity of tumour localisation using intact antibodies was poor. It has been reported that affinity and specificity of binding *in vitro* do not necessarily predict selectivity and specificity of uptake *in vivo* (Mann *et al.*, 1984; Sakahara *et al.*, 1988). Since antibody binding to Fc receptors may contribute to the low selectivity of antibody accumulation in tumours, improved tumour-to-background ratios can be achieved by the use of F(ab')$_2$ or Fab fragments. In several studies, the use of such fragments resulted in images superior to those from intact antibodies (Buchegger *et al.*, 1983; Wahl *et al.*, 1983; Herlyn *et al.*, 1983; Durbin *et al.*, 1988). Fab fragments were omitted from this study since they are reported to be inferior to F(ab')$_2$ fragments and unsuitable for use in patients due to their low absolute tumour uptake, very high clearance rates, and *in vivo* instability (Wahl *et al.*, 1983; Durbin *et al.*, 1988).

The production of F(ab')$_2$ fragments, however, may be hampered because of the unpredictability of pepsin digestion (Mather *et al.*, 1987; Lamoyi & Nisonoff, 1983; Durbin *et al.*, 1988). In our hands, pepsin digestion of the antibodies proved satisfactory for 7E8 and OKT3 only. The latter antibody is of the IgG2a subclass, but blood clearance rates for 7E8 F(ab')$_2$ and OKT3 F(ab')$_2$ were similar and no preferential uptake in any tissue was seen for the OKT3 F(ab')$_2$ fragment, making it suitable as a non-specific control for 7E8 F(ab')$_2$.

Although labelled 7E8 and its F(ab')$_2$ fragment had comparable affinities for purified PLAP and for PLAP *in vitro* on the cell surface, the F(ab')$_2$ fragments had quite different biodistribution and tumour localisation properties as compared with results for intact antibody. In accordance with earlier reports (Buchsbaum *et al.*, 1988; Durbin *et al.*, 1988), we found that F(ab')$_2$ fragments were cleared more rapidly from blood than intact antibody, with a catabolic phase half-life of 4 to 5 h, as compared with 62 to 107 h for intact antibodies. Elimination rates of 7E8 F(ab')$_2$ fragments from control tumour and non-tumour tissue were much higher than from the PLAP-positive tumour. The uptake of 7E8 F(ab')$_2$ fragments was only elevated in the kidney, which is known as a major site of fragment catabolism in mice (Covell *et al.*, 1986). It has been reported that F(ab')$_2$ fragments have reduced absolute tumour uptake compared with intact IgG (Buchegger *et al.*, 1983; Blumenthal *et al.*, 1989; Sharkey *et al.*, 1990). However, we found that peak levels of F(ab')$_2$ fragments in antigen-expressing tumour (at about 7.5% of injected dose/g, 2 h after injection) were even higher than those found for intact antibodies. Thereafter, the level of fragments declined faster than that of intact antibody. It has been thought that intra-tumour catabolism of fragments proceeds faster than catabolism of intact antibodies (Pimm & Baldwin, 1987*a*; Endo *et al.*, 1987; Pimm *et al.*, 1989).

When selective tumour localisation was expressed as tumour to non-tumour tissue ratio, 7E8 F(ab')$_2$ fragments scored about 12 times better than intact 7E8 and seven times better than intact 17E3 (mean geometric SI was 34.5 at 46 h). In addition, tumour localisation by 7E8 F(ab')$_2$ fragments was highly specific with a localisation index of 29.0 at 46 h. As expected, excellent imaging of MO4 1-4 tumours with 7E8 F(ab')$_2$ fragments was obtained upon gamma camera recording. These results complement the earlier findings obtained for localisation of HeLa cell tumours via the PLAP/anti-PLAP system with polyclonal or monoclonal antibodies (Jeppsson *et al.*, 1984) and antibody fragments (Durbin *et al.*, 1988; Stigbrand *et al.*, 1989), although the latter authors report a higher efficiency for intact antibodies than for F(ab')$_2$ fragments.

In conclusion, we can state that (1) recombinant MO4 1-4 tumours grown in the thighs of nude mice are a useful *in vivo* model for tumour immuno-targeting due to their stable expression of PLAP on the plasma membrane; (2) it is possible to discriminate PLAP-expressing tumours in nude mice, using a $^{125}$I-labelled monoclonal antibody with a rather low affinity for PLAP (17E3); and (3) excellent selective and specific immunolocalisation and high imaging efficiency of the same tumours is achieved upon injection of F(ab')$_2$ fragments of the high affinity 7E8 antibody which, when injected as intact antibody, did not result in meaningful tumour localisation.

This work was supported by grants from the 'Vereniging voor Kankerbestrijding', and 'Kankerfond; of the ASLK, and the 'IWONL (VL 162)'. We wish to acknowledge Dr M. Mareel for providing us the MO4 and 5583-S cells and M. Goergen for transfecting the MO4 cells. We also wish to thank Dr J. Chan for the anti-CEA monoclonal antibody. Dr A. Vrancken and M. Lambrechts are acknowledged for their expert assistance in radionuclide scanning and M. Elseviers for her contribution in the mathematical analyses. The excellent secretarial work of E. Van Hout is gratefully appreciated.

### References

ANDREW, S.M., PIMM, M.V., PERKINS, A.C. & BALDWIN, R.W. (1986). Comparative imaging and biodistribution studies with an anti-CEA monoclonal antibody and its F(ab)2 and F(ab) fragments in mice with colon carcinoma xenografts. *Eur. J. Nucl. Med.*, **12**, 169.

BALE, W.F., CONTRERAS, M.A. & GRADY, E.D. (1980). Factors influencing localization of labeled antibodies in tumors. *Cancer Res.*, **40**, 2965.

BILLIAU, A., SOBIS, H., EYSSEN, H. & VANDEN BERGHE, N. (1973). Non-infectious intracysternal A-type particles in a sarcoma-positive, leukemia-negative mouse cell line transformed by murine sarcoma virus (MSV). *Arch. Gesamt. Virusforsch*, **43**, 345.

BLUMENTHAL, R.D., SHARKEY, R.M., KASHI, R. & GOLDENBERG, D.M. (1989). Comparison of therapeutic efficacy and host toxicity of two different 131-labeled antibodies and their fragments in the GW-39 colonic cancer xenograft model. *Int. J. Cancer*, **44**, 292.

BUCHEGGER, F., HASKELL, C.M., SCHREYER, M. & 4 others (1983). Radiolabeled fragments of monoclonal antibodies against carcinoembryonic antigen for localization of human colon carcinoma grafted into nude mice. *J. Exp. Med.*, **158**, 413.

BUCHEGGER, F., MACH, J.P., LEONNARD, P. & CARREL, S. (1986). Selective tumor localization of radiolabeled anti-human melanoma monoclonal antibody fragment demonstrated in the nude mouse model. *Cancer*, **58**, 655.

BUCHSBAUM, D.J., SINKULE, J.A., STITES, M.S. & 6 others (1988). Localization and imaging with radioiodine-labeled monoclonal antibodies in a xenogeneic tumor model for human B-cell lymphoma. *Cancer Res.*, **48**, 2475.

COVELL, D.G., BARBET, J., HOLTON, O.D., BLACK, C.D.V., PARKER, R.J. & WEINSTEIN, J.N. (1986). Pharmacokinetics of monoclonal immunoglobulin $G_1$, F(ab')$_2$ and Fab' in mice. *Cancer Res.*, **46**, 3969.

CHANG, C.H., ANGELLIS, D. & FISHMAN, W.H. (1980). Presence of the rare D-variant heat stable placental-type alkaline phosphatase in normal human testis. *Cancer Res.*, **40**, 1506.

DE BROE, M.E & POLLET, D.E. (1988). Multicenter evaluation of human placental alkaline phosphatase as a possible tumour ,associated antigen in serum. *Clin. Chem.*, **34**, 1995.

DIXON, W.J. (1988). BMDP Statistical Software Manual. Dixon, W.J. (ed.) University of California: Los Angeles, CA.

DURBIN, H., MILLIGAN, E.M., MATHER, S.J., TUCKER, D.F., RAYMOND, R. & BODMER, W.F. (1988). Monoclonal antibodies to placental alkaline phosphatase: preclinical evaluation in a human xenograft tumour model of F(ab')$_2$ and Fab fragments. *Int. J. Cancer*, (Suppl), **2**, 59.

ENDO, K., KAMMA, H. & OGATA, T. (1987). Radiolocalization of xenografted human lung cancer with monoclonal antibody 8 in nude mice. *Cancer Res.*, **47**, 5427.

ECCLES, S.A., PURVIES, H.P., STYLES, J.M., HOBBS, S.M. & DEAN, C.J. (1989). Pharmacokinetic studies of radiolabeled rat monoclonal antibodies recognising syngeneic sarcoma antigens. *Cancer Immunol. Immunother.*, **30**, 5.

GOLDSTEIN, D.J., ROGERS, C. & HARRIS, H. (1982). A search for trace expression of placental-like alkaline phosphatase in nonmalignant human tissues: demonstration of its occurrence in lung, cervix, testis and thymus. *Clin. Chim. Acta*, **125**, 63.

GOSSRAU, R. (1978). Azoindoxylverfahren zum Hydrolasennachweiss. IV. Zur Eignung verschiedener Diazoniumsalze. *Histochemistry*, **57**, 323.

GREENWOOD, F.C., HUNTER, W.M. & GLOVER, J.S. (1963). The preparation of $^{131}$I-labeled human growth hormone of high specific radioactivity. *Biochem. J.*, **89**, 114.

HEDIN, A., WAHREN, B. & HAMMARSTRÖM, S. (1982). Tumor localization of cea-containing human tumors in nude mice by means of monoclonal anti-cea antibodies. *Int. J. Cancer*, **30**, 547.

HENDRIX, P.G., HOYLAERTS, M.F., NOUWEN, E.J. & DE BROE, M.E. (1990). Enzyme immunoassay of human placental and germ-cell alkaline phosphatase in serum. *Clin. Chem.*, **36**, 1793.

HERYLN, D., POWE, J., ALAVI, A. & 5 others (1983). Radioimmunodetection of human tumor xenografts by monoclonal antibodies. *Cancer Res.*, **43**, 2731.

JEPPSSON, A., WAHREN, B., MILLAN, J.L. & STIGBRAND, T. (1984). Tumor and cellular localization by use of monoclonal and polyclonal antibodies to placental alkaline phosphatase. *Br. J. Cancer*, **49**, 123.

KOSHIDA, K. & WAHREN, B. (1990). Placental-like alkaline phosphatase in seminoma. *Urol. Res.*, **18**, 87.

LAEMMLI, U.K. (1970). Cleavage of structural proteins during the assembly of the head of bacteriophage T4. *Nature*, **227**, 680.

LAMOYI, E. & NISONOFF, A. (1983). Preparation of F(ab')$_2$ fragments from mouse IgG of various subclasses. *J. Immunol. Methods*, **56**, 235.

MANN, B.D., COHEN, M.B., SAXTON, R.E. & 7 others (1984). Imaging of human tumour xenografts in nude mice with radiolabeled monoclonal antibodies. *Cancer*, **54**, 1318.

MAREEL, M., DE RIDDER, L., DE BRABANDER, M. & VAKAERT, L. (1975). Characterization of spontaneous, chemical and viral transformants of a C3H/3T3-type mouse cell by a transplantation into young chick blastoderms. *J. Natl Cancer Inst.*, **54**, 923.

MATHER, S.J., DURBIN, H. & TAYLOR-PAPADIMITRIOU, J. (1987). Identification oif immunoreactive monoclonal antibody fragments for improved immunoscintigraphy. *J. Immunol. Methods*, **96**, 255.

MATZKU, S., KIRCHGESSNER, H., DIPPOLD, W.G. & BRÜGGEN, J. (1985). Immuno-reactivity of monoclonal anti-melanoma antibodies in relation to the amount of radioactive iodine substituted to the antibody molecular. *Eur. J. Nucl. Med.*, **11**, 260.

MEYVISCH, C. & MAREEL, M. (1982). Influence of implantation site of MO4 cell aggregates on the formation of metastases. *Invasion Metastasis*, **2**, 51.

MILLAN, J.L. (1985). Molecular cloning and sequence analysis of human placental alkaline phosphatase. *L . Biol. Chem.*, **261**, 3112.

MILLAN, J.L. & STIGBRAND, T. (1983). Antigenic determinants of human placental and testicular placental-like alkaline phosphatase as mapped by monoclonal antibodies. *Eur. J. Biochem.*, **136**, 1.

MOSHAKIS, V., MCILHINNEY, R.A.J., RAGHAVAN, D. & NEVILLE, A.M. (1981). Monoclonal antibodies to detect human tumors: an experimental approach. *J. Clin. Pathol.*, **34**, 314.

NOUWEN, E.J., POLLET, D.E., EERDEKENS, M.W., HENDRIX, P.G., BRIERS, T.W. & DE BROE, M.E. (1986). Immunohistochemical localization of placental alkaline phosphatase, carcinoembryonic antigen and cancer antigen 125 in the normal and neoplastic human lung. *Cancer Res.*, **46**, 866.

NOUWEN, E.J., HENDRIX, P.G., DAUWE, S., EERDEKENS, M.W. & DE BROE, M.E. (1987). Tumor markers in the human ovary and its neoplasms: a comparative immunohistochemical study. *Am. J. Pathol.*, **126**, 230.

PIMM, M.V. & BALDWIN, R.W. (1985). The influence of increasing specific activity on the immunological reactivity of 131-labelled anti-tumor monoclonal antibody. *IRCS Med. Sci.*, **13**, 497.

PIMM, M.V. & BALDWIN, R.W. (1987*a*). Comparative biodistributions and rates of catabolism of radiolabelled anti-CEA monoclonal antibody and control immunoglobulin in nude mice with human tumour xenografts showing specific antibody localization. *Eur. J. Nucl. Med.*, **13**, 258.

PIMM, M.V. & BALDWIN, R.W. (1987*b*). Comparative tumour localization properties of radiolabelled monoclonal antibody preparations of defined immunoreactivities. *Eur. J. Nucl. Med.*, **13**, 348.

PIMM, M.W., PERKINS, A.C. & BALDWIN, R.W. (1987). Diverse characteristics of $^{111}$in labelled anti-CEA monoclonal antibodies for tumour immunoscinitigraphy: radiolabelling, biodistribution and imaging studies in mice with human tumour xenografts. *Eur. J. Nucl. Med.*, **12**, 515.

PIMM, M.V., ANDREW, S.M. & BALDWIN, R.W. (1989). Blood and tissue kinetics of radiolabelled anti-CEA monoclonal antibody and $F(ab)_2$ and Fab fragments in nude mice with human tumour xenografts: implications for tumour imaging and radioimmunotherapy. *Nucl. Med. Comm.*, **10**, 585.

ROBSON, E.B. & HARRIS, H. (1965). Genetics of the alkaline phosphatase polymorfism of the human placentae. *Nature*, **207**, 1257.

SAKAHARA, H., ENDO, K., KOIZUMI, M. & 11 others (1988). Relationship between *in vitro* binding activity and *in vivo* tumor accumulation of radiolabeled monoclonal antibodies. *J. Nucl. Med.*, **29**, 235.

SHARKEY, R.M., GOLD, D.V., ANINIPOT, R. & 6 others (1990). Comparison of tumor targeting in nude mice by murine monoclonal antibodies directed against different human colorectal cancer antigens. *Cancer Res.*, (Suppl.) **50**, 828s.

STIGBRAND, T., HIETALA, S.O., JOHANSSON, B., MAKIYA, R., RIKLUND, K. & EKELUND, L. (1989). Tumour radioimmunolocalization in nude mice by use of antiplacental alkaline phosphatase monoclonal antibodies. *Tumor Biol.*, **10**, 243.

VERSTIJNEN, C.P., ARENDS, J.W., MOEKERK, P.T.M. & 4 others (1987). The establishment and characterization of two new cell lines derived from a single human colonic adenocarcinoma. *Virchows Arch. B.*, **53**, 191.

WAHL, R.L., PARKER, C.W. & PHILPOTT, G.W. (1983). Improved radioimaging and tumor localization with monoclonal $F(ab')_2$. *J. Nucl. Med.*, **24**, 316.